# Disruption of Neuronal Autophagy by Infected Microglia Results in Neurodegeneration

**DOI:** 10.1371/journal.pone.0002906

**Published:** 2008-08-06

**Authors:** Mehrdad Alirezaei, William B. Kiosses, Claudia T. Flynn, Nathan R. Brady, Howard S. Fox

**Affiliations:** 1 Molecular and Integrative Neurosciences Department, The Scripps Research Institute, La Jolla, California, United States of America; 2 Core Microscopy Facility, The Scripps Research Institute, La Jolla, California, United States of America; 3 Deutsches Krebsforschungszentrum and BIOQUANT, Heidelberg, Germany; Emory University, United States of America

## Abstract

There is compelling evidence to support the idea that autophagy has a protective function in neurons and its disruption results in neurodegenerative disorders. Neuronal damage is well-documented in the brains of HIV-infected individuals, and evidence of inflammation, oxidative stress, damage to synaptic and dendritic structures, and neuronal loss are present in the brains of those with HIV-associated dementia. We investigated the role of autophagy in microglia-induced neurotoxicity in primary rodent neurons, primate and human models. We demonstrate here that products of simian immunodeficiency virus (SIV)-infected microglia inhibit neuronal autophagy, resulting in decreased neuronal survival. Quantitative analysis of autophagy vacuole numbers in rat primary neurons revealed a striking loss from the processes. Assessment of multiple biochemical markers of autophagic activity confirmed the inhibition of autophagy in neurons. Importantly, autophagy could be induced in neurons through rapamycin treatment, and such treatment conferred significant protection to neurons. Two major mediators of HIV-induced neurotoxicity, tumor necrosis factor-α and glutamate, had similar effects on reducing autophagy in neurons. The mRNA level of p62 was increased in the brain in SIV encephalitis and as well as in brains from individuals with HIV dementia, and abnormal neuronal p62 dot structures immunoreactivity was present and had a similar pattern with abnormal ubiquitinylated proteins. Taken together, these results identify that induction of deficits in autophagy is a significant mechanism for neurodegenerative processes that arise from glial, as opposed to neuronal, sources, and that the maintenance of autophagy may have a pivotal role in neuroprotection in the setting of HIV infection.

## Introduction

Macroautophagy (referred here as autophagy) is a highly inducible mechanism upon stress or injury in cells, in which a small proportion of cytoplasm, including organelles, is engulfed within double membrane vacuoles, known as autophagosomes or autophagy vacuoles (AV), and then degraded by fusion with lysosomes [1], [2]. In addition to this inductive component, constitutive autophagy has been found to be an indispensable mechanism in terminally-differentiated cells such as neurons in order clear up damaged long-lived nuclear and cytosolic proteins or organelles. Autophagy is especially important where the ubiquitin-proteasome apparatus is inefficient because of large molecule size or its unselectively [3]–[5].

To date, several autophagy related genes (*Atg*) and their respective proteins are known to directly regulate the formation of AV in mammalian cells [2], [6]. Among these proteins, it is known that the covalently-bound complex of Atg12-Atg5 regulates the elongation of AV isolation membrane known as phagophore [7], [8]. In addition, Atg12-Atg5 is an ubiquitin E3-like enzyme that conjugates the microtubule-associated protein 1 light chain 3 (LC3, also known as Atg8) to phosphatydilethanolamine, known as the conversion of LC3-I to LC3-II [9], [10]. LC3-II binds to the membrane of AV and regulates their formation in cells. To date, LC3-II is the best identified mammalian protein species that specifically associates with AV membranes [10].

Recent studies have demonstrated that the suppression of constitutive autophagy in neurons results in severe neurodegenerative disorders [1], [5]. The selective genetic ablation of *Atg5* or *Atg7* in neurons leads to motor and behavioral deficits associated with neuronal loss in the hippocampus, cerebral and cerebellar cortices [1], [5], [11], [12]. This clinical pathology observed in neuron-specific autophagy-deficient mice parallels findings in patients with neurodegenerative disorders [1]. These experiments clearly demonstrate that autophagy is constitutively active in neurons and is required for neuronal survival. In addition, the genetic disruption of autophagy greatly impaired the degradation of ubiquitin-protein aggregates, resulting in an accumulation of ubiquitinated proteins in neurons [11], [12]. Generally, short-lived proteins are degraded *via* the ubiquitin-proteasome pathway, but when there is proteasome dysfunction, autophagy can can serve to degrade these proteins, as it does constitutively for the long lived proteins [4].

Interestingly, the LC3-binding protein p62 (also known as sequestosome 1), was initially identified as a protein that is induced as a response to the oxygen radical stress or to the inhibition of proteasomal activity [13]–[15]. Indeed, recent work has shown that p62 couples polyubiquitinated protein aggregates to the autophagy machinery *via* LC3, and facilitates the clearance of aggregates resulting reduced toxicity [16], [17]. Thus, in addition to LC3, p62 can also be employed as an autophagic marker as it is degraded by autophagic machinery, and there is a correlation between inhibition of autophagy and increased levels of p62 [16]–[19].

HIV- or Simian Immunodeficiency Virus (SIV)-associated central nervous system (CNS) disease is an acquired form of neurodegeneration resulting from the production of molecules from activated and infected macrophages/microglia in the brain [20], [21]. Such non-neuronal sources of neurodegeneration, referred to as non-cell autonomous neurodegenerative processes are becoming increasing recognized to extend beyond infectious diseases and may initiate and/or contribute to the progression of a variety of neurodegenerative disorders [22]. Neuronal damage is well-documented in the brains of HIV-infected individuals, and evidence of inflammation, oxidative stress, damage to synaptic and dendritic structures, and neuronal loss are present in the brains of those with HIV-associated dementia (HAD) [23]–[26]. Intriguingly, ubiquitin protein deposits are abundant in neurons in those with HAD [27].

Using the well-characterized neurodegenerative activity of products of SIV- or HIV-infected myeloid cells on neurons [28], [29], we investigated the role of autophagy in neurodegeneration in individual neurons primary rat cortical cultures. We evaluated the number and location of AV using a high sensitivity approach consisting of a combination of confocal microscopy and three-dimensional reconstruction imaging software. Primary neurons, expressing LC3 marked by green fluorescent protein (GFP), were assessed for the presence and number of AV (as marked by GFP-LC3) in both soma and processes. Images were acquired and analyzed from neurons exposed to the supernatant of SIV-infected microglia, derived from primary cultures of microglia from animals with SIV encephalitis (SIVE). We then confirmed the imaging results with the use of diverse biochemical markers and chemical modifiers of autophagy, revealing the ability of glial products to inhibit autophagy and decrease neuronal survival. Changes in p62 mRNA levels, determined in a primate model (SIVE and uninfected brains) and neuronal p62 protein distribution and content, detected by immunohistochemistry in monkey brain tissues and HAD brains, support the *in vivo* relevance of these findings.

## Results

### Three-dimensional modeling of primary neurons for AV quantification

**Figure 1:**
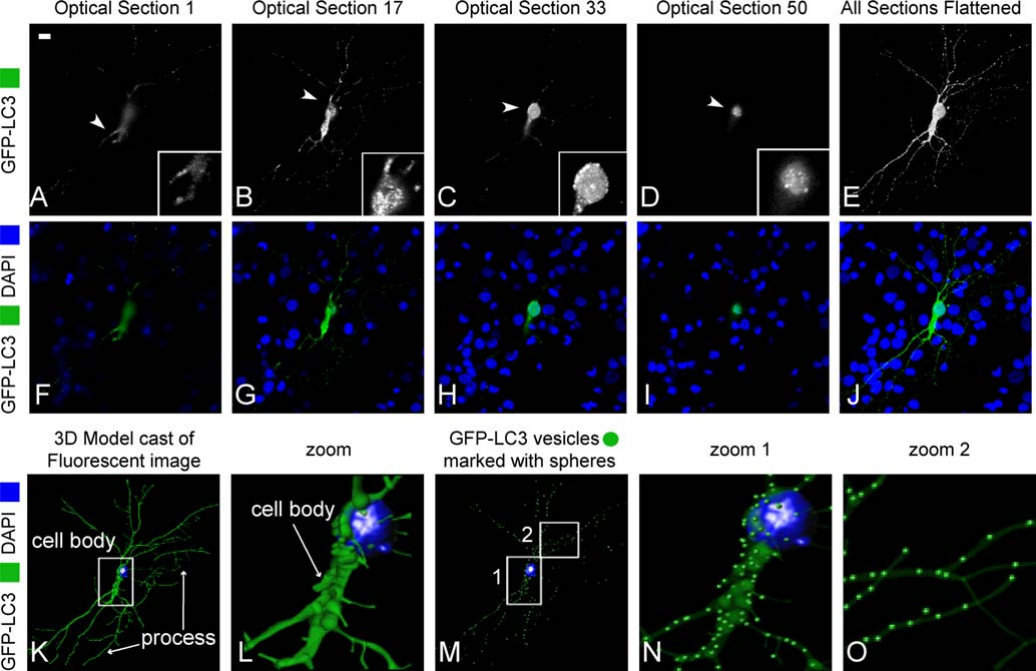
Confocal imaging and three-dimensional model reconstruction of neuron transfected with GFP-LC3. (A–J) Confocal images of different *z* sections (0.2 µm of thickness) of a neuron transfected with GFP-LC3 (A–D) and all sections are flattened (E). Higher magnification images of the area outlined in white are shown in the inserts (A–D). Different sections of images of transfected neuron with GFP-LC3 (green) merged with DAPI (blue). Scale bar, 20 µm. (K–O) Three-dimensional reconstruction from serial sections using IMARIS software, cell body and process (K) and higher magnification of the zoomed area outlined in white (K-I). Reconstructed 3D image of the same neuron using IMARIS, which evaluates AV localization in the reconstructed 3D image (M–O). Enlarged areas of neuron revealed the presence of AV in soma and neurites (N–O).

Although there are indications that autophagy is active in developing primary neuronal cultures [3], [29], techniques for detecting autophagy in neurons have to date been qualitative. As a first objective we established a method to quantitatively determine the number of AV in primary neuronal cultures, both in the soma and processes. Primary neuronal cultures were transfected with GFP-LC3 to allow visualization of AV. Conditions of low neuronal transfection efficiency (<2%) were used in order to observe the soma and processes of single GFP-LC3 expressing neurons within the matrix of other neurons. To quantify the spatial and temporal distribution of AV we acquired confocal *z*-stacks for transfected neurons (Figures 1A–1J, 0.2 µm step size, approximately 150 images acquired per cell). From these data sets we were able to determine the 3D structure of the soma and processes, as well as the number and distribution of GFP-LC3-labelled AV (Figures 1K–1O). This imaging approach allowed for the quantitative investigation of AV content and localization in different conditions in order to understand their potential changes in our *in vitro* model of SIV microglia supernatant inducing neuronal cell death.

### Microglia supernatant from SIV-infected rhesus monkeys inhibits the generation of AV in primary neurons in a rapamycin-reversible manner

**Figure 2:**
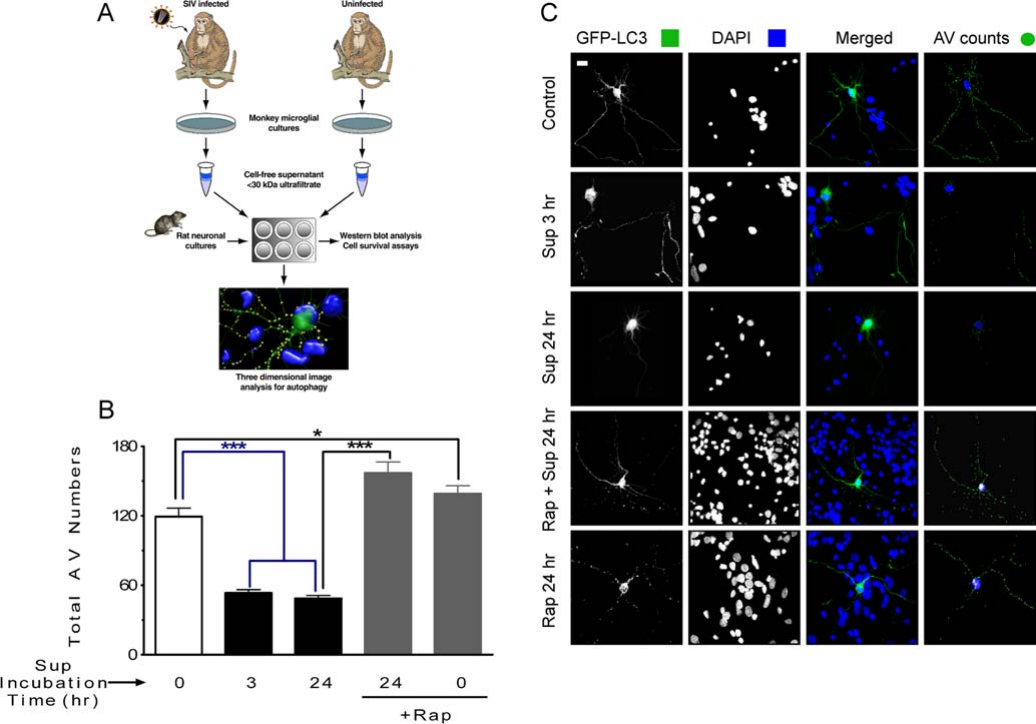
*In vitro* analysis of the impact of SIV-infected microglia supernatant on autophagy in primary neurons. (A) Model of experimental design, as detailed in the Materials and Methods. (B) Total AV counting results show that a significant drop in the number of AV occurs in neurons exposed to SIV-infected microglia supernatant for 3 or 24 hr. Total number of AV are significantly increased after the pretreatment with rapamycin followed by SIV-infected microglia supernatant (Sup) exposure for 24 hr. *** *P*<0.001, * *P*<0.05 for *n* = 6 experiments. All values are mean±SEM. AV counting using 3D model reconstruction for neurons exposed to different treatments. (C) Flattened images of multi-stack confocal optical slices of neurons transfected with GFP-LC3 (green) to label AV, and DAPI (blue) to label the nucleus. The five panels display a typical sample image of a neuron under control conditions as well as 3 hr and 24 hr exposure to SIV-infected microglia supernatant or pretreated with rapamycin prior the exposure to SIV-infected microglia supernatant. The right hand panels show a snap shot of a 3D outline of the neuron with AV marked as green spheres. Scale bar, 20 µm.

Subsequently, to examine the role of autophagy in SIV mediated neuronal cell death, we treated GFP-LC3-transfected primary rat neurons, as described above, with supernatants from microglia/macrophages of SIV-infected or uninfected monkeys and determined the total number of AV. Separate monkey microglia cultures were derived from cultures from infected and uninfected animals, and the culture supernatants then subjected to ultrafiltration to isolate molecules less than 30 kDa for use as microglia-derived factors to treat primary neurons (Figure 2A). Under control conditions, the mean number of AV in primary control neurons was 119.3±7.4 (Figure 2B), likely representing the constitutive autophagy activity necessary for neuronal survival. In many cell types autophagy is induced as a starvation response, activated to provide sources of energy. This induction occurs through inhibition of the mammalian target of rapamycin (mTOR), which negatively regulates the autophagy pathway in cells, and can be mimicked by treatment with the mTOR inhibitor rapamycin [30]. However, perhaps given the priority of the brain in obtaining nutrients, autophagy is not strongly induced in CNS neurons as part of the starvation response [31]. We found that neurons treated for 24 hr with rapamycin exhibited a slight but significant increase in AV number (Figures 2B and 2C, 139.2±6.8), indicating that autophagy can be pharmacologically activated in our experimental model.

We next examined whether the supernatant from SIV-infected microglia had an effect on the number of AV. Exposure resulted in a significant reduction in the number of AV after 3 hr of exposure (Figures 2B and 2C, 53.5±2.8), which remained low following 24 hr of exposure (Figures 2B and 2C, 48.6±2.5). Neurons treated with supernatant from microglia cultured from uninfected monkeys exhibited AV numbers indistinguishable statistically from untreated controls (150±21.6). The observed decrease in AV content indicated either (i) the rate of AV degradation was higher than the rate of AV formation, (ii) AV formation was significantly inhibited, or (iii) the loss of neurites contribute to the loss of AV. To help address this, we then determined whether rapamycin could prevent the decrease of AV in neurons induced by the microglia supernatant. Neurons were pretreated with rapamycin followed by exposure to the supernatant for 24 hr, resulting in complete protection from the decrease in number of AV (Figures 2B and 2C, 157±9.6). Taken together, these data indicate that the supernatant from SIV-infected microglia inhibited the induction of autophagy in primary neurons, and this effect was reversible with rapamycin treatment.

### Altered subcellular distribution of AV and loss of neurites

**Figure 3:**
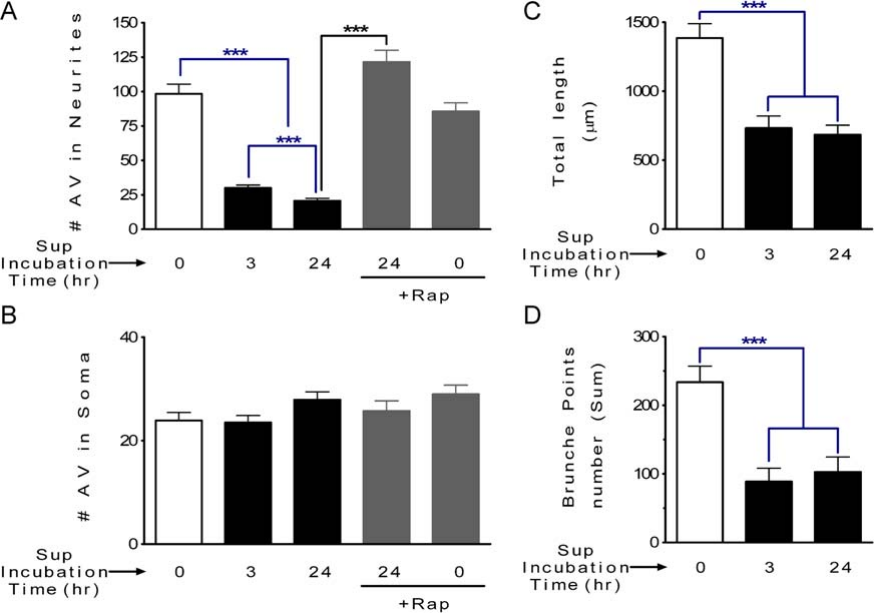
Process length and AV distribution in neurons exposed to different treatments. (A) The number of AV in neurites decreased significantly in neurons exposed to the SIV-infected microglia supernatant for 3 or 24 hr, however; pretreatment with rapamycin blocked this effect. *** *P*<0.001, for *n* = 6 experiments. Scale bar, 20 µm. (B) The AV number is unchanged in neuronal soma after exposure to the SIV-infected microglia supernatant for 3 or 24 hr. (C and D) Both the total length of neuronal processes (C) and number of brunch points (D) are decreased after exposure to after SIV-infected microglia supernatant treatment. *** *P*<0.001, for *n* = 6 experiments. All values are mean±SEM.

Next, we determined if the subcellular AV distribution was altered after exposure to the microglia supernatant. By differentiating the limit of the neuronal cell body from the processes, we were able to quantify the distribution of AV in the cell body and in the processes of individual neurons. As shown in Figure 3A, the number of cellular AV decreased significantly in processes of individual neurons after exposure to microglia supernatant for 3 or 24 hr (Figure 3A) whereas at the same time points the AV number was unchanged in the soma of neurons (Figure 3B). These data indicate that in addition to an inhibitory effect, the exposure of neurons to SIV-infected microglia supernatant treatment altered the subcellular distribution of AV. Rapamycin stimulation of autophagy, under control conditions, did not alter the subcellular distribution of AV (Figures 3A and 3B). Moreover, rapamycin pretreatment, in the presence SIV-infected microglia supernatant, prevented the alterations in distribution of AV between the soma and neurites (Figures 3A and 3B).

We observed a dramatic reduction of AV number in the processes of neurons exposed to the SIV-infected microglia supernatant, therefore it was necessary to determine if process integrity was compromised. We thus determined process length and number of process branch points per cell from high-resolution confocal *z*-stacks of neurons labeled with GFP-LC3. Three-dimensional image analysis revealed that both the total process length, as well as the number of branch points, was significantly reduced in neurons treated with SIV-infected microglia supernatant after 3 or 24 hr (Figures 3C and 3D). Therefore the SIV microglia supernatant triggered the loss of the neuronal processes, and in the remaining neurites, there are decreased numbers of AV.

### Biochemical markers of autophagy in neuronal cultures exposed to SIV microglia supernatant

**Figure 4:**
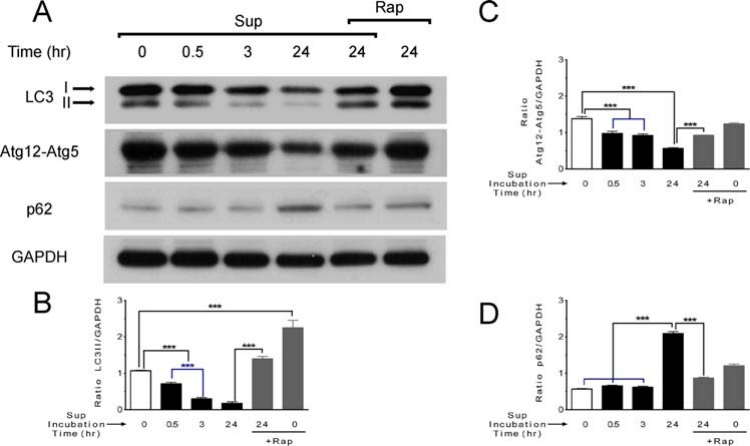
Western blot analysis of autophagy related proteins. (A) The LC3-II level is reduced after 3 or 24 hr exposure to SIV-infected microglia supernatant and this decrease effect is blocked when is pretreated with rapamycin (2 µM). The protein level of elongation complex Atg12-Atg5 is also reduced, and again the effect is blocked in the presence of rapamycin. The protein level of p62 is increased for similar conditions with SIV-infected microglia supernatant, and the increase is blocked in the presence of rapamycin. GAPDH protein was used in these experiments as the loading control. One representative experiment of *n* = 4 is shown. (B, C and D) Ratios between LC3-II, Atg12-Atg5, p62, respectively, normalized to GAPDH. Data are reported as mean±SEM (*n* = 4). *** *P*<0.001.

We further investigated the observed impairment of neuronal autophagy by SIV-infected microglia supernatant by Western blot detection of different parameters of autophagic activity. The conversion of LC-I to LC3-II is correlated with the number of AV, and can be assessed by immunoblotting [10], [32], [33]. A second approach to examine potential impairment of autophagy is measuring the protein level of the conjugate Atg12-Atg5 that drives the elongation step of autophagy [8]. A third alternative technique for identifying the inhibition of autophagy is measuring p62 protein upregulation [16], [34]. Its level therefore can be used as an index of autophagic degradative capacity. We used all three of these methods to examine the potential impairment of autophagy induced by the SIV-infected microglia supernatant. Similar to three dimensional imaging results (Figures 2 and 3), Western blot detection of LC3 revealed a time-dependent decrease in LC3-II levels, indicating that neuronal AV numbers were significantly decreased in the presence of the SIV-infected microglia supernatant in a time-dependent manner (Figure 4A). Detection of the Atg12-Atg5 conjugate, revealed a time-dependent decrease in conjugate formation (Figure 4A), evidencing inhibition to autophagy. Examination of p62 protein levels revealed a significant increase after 24 hr exposure to the microglia supernatant (Figure 4A). Importantly, rapamycin increased both LC3-II and Atg5-Atg12 conjugate levels, in both control cells and cells exposed to SIV-infected microglia supernatant (Figure 4A), consistent with impairment of autophagy. Moreover, rapamycin decreased p62 levels in neurons treated with SIV-infected microglia supernatant to control levels. The optical density of bands was measured using imageJ software and the associated graphs were presented (Figures 4B–4D). Control supernatant from uninfected monkeys did not affect the LC3-II level for 3 and 24 hr exposure time (data not shown).

### Impact of effector molecules on neuronal autophagy

**Figure 5:**
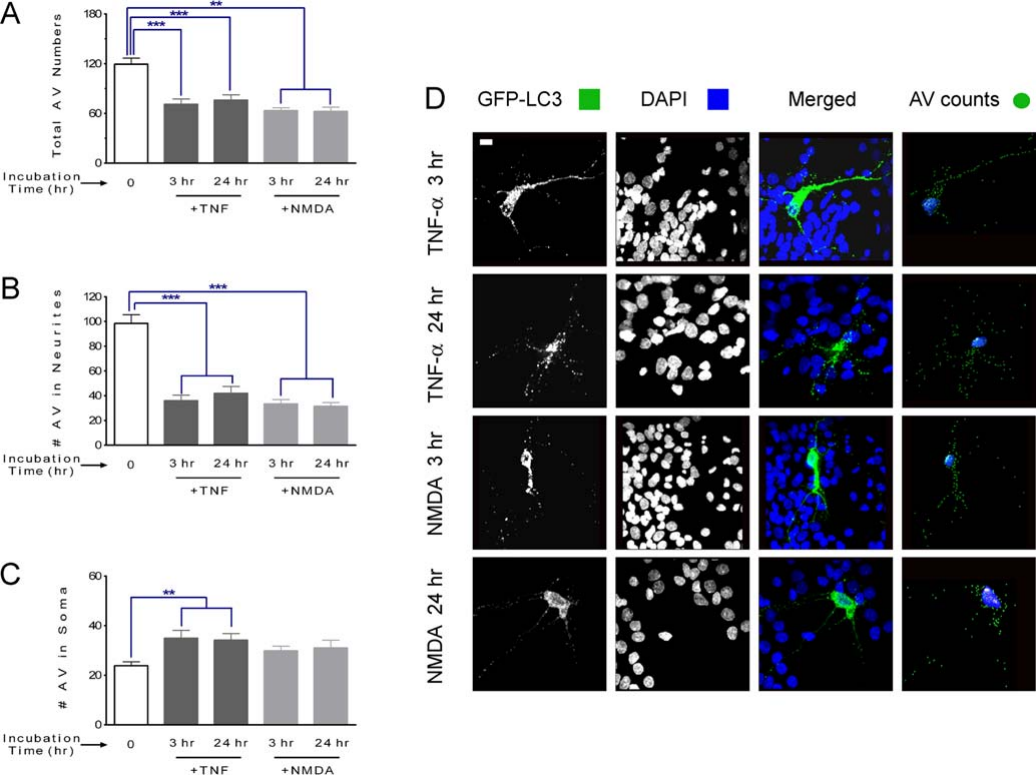
AV counting and distribution for neurons exposed to different treatments. (A) Total AV counting results show that a significant drop occurs in neurons exposed to TNF-α or NMDA for 3 or 24 hr. *** *P*<0.001, ** *P*<0.01 for *n* = 6 experiments. All values are mean±SEM. (B and C) Distribution of AV number in soma and neurites respectively. (B) The number of AV in neurites decreased significantly in neurons exposed to both TNF-α or NMDA for 3 or 24 hr. *** *P*<0.001, for *n* = 6 experiments. (C) The AV number in soma is increased in neurons after exposure to TNF-α for 3 or 24 hr, however it remained unaffected in neurons after exposure to NMDA. ** *P*<0.01 for *n* = 6 experiments. (D) Flattened images of multi-stack confocal optical slices of neurons transfected with GFP-LC3 (green) to label AV, and DAPI (blue) to label the nucleus. transfected with GFP-LC3 (green) to label AV, and DAPI (blue) to label the nucleus. The four panels display a typical sample image of a neuron under 3 hr and 24 hr exposure to TNF-α or NMDA at 25 ng/ml and 35 µM respectively. Scale bar, 20 µm.

TNF-α and glutamate are major pro-inflammatory and excitotoxic factors associated with neuronal death in HAD patients and are potential candidate neurotoxins present in the SIV-infected microglia supernatant [21]. To assess whether these agents can themselves affect autophagy, we studied, separately, the effect of TNF-α and the excitotoxic glutamate agonist *N*-methyl-d-aspartate acid (NMDA) on AV in neurons. The total AV number decreased significantly in neurons exposed to either TNF-α or NMDA (Figures 5A and 5D). Both molecules had their main effect in by reducing the number of AV in neurites (Figures 5B and 5D) whereas TNF-α led to a small but significant increase in AV in the soma (Figures 5C and 5D).

### Role of autophagy in neuronal survival

**Figure 6:**
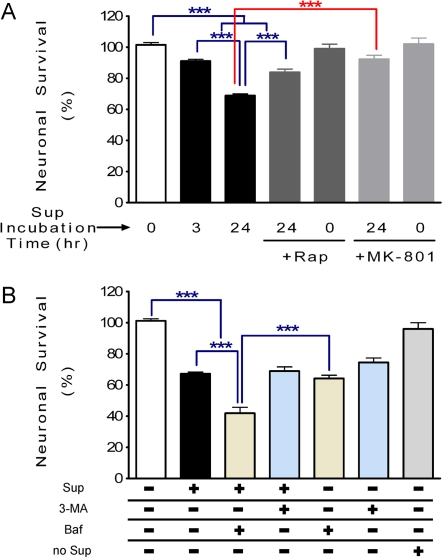
Neuronal survival after exposure to SIV-infected microglia supernatant in the presence of different autophagy related drugs. (A). Neuronal survival tested by MTT. There are significant decreases of neuronal survival after exposure to SIV-infected microglia supernatant for 3 or 24 hr, which is significantly abrogated in the presence of rapamycin. Rapamycin alone has no effect. MK-801 (100 µM) also prevented the neurotoxicity induced by SIV-infected microglia supernatant. *** *P*<0.001, for *n* = 6 experiments. (B) Neuronal survival was examined with MTT in cultures exposed to SIV-infected microglia supernatant for 24 hr and in the presence or absence of different drugs such as 3-MA (1 mM) or Baf (100 nM). There is no significant difference between cells exposed to SIV-infected microglia supernatant and a pretreatment with 3-MA followed by exposure to SIV-infected microglia supernatant. However, there is a significant decrease of neuronal survival exposed to SIV-infected microglia supernatant when cells are pretreated with Baf. The SIV-infected microglia supernatant from no infected monkey (no Sup) doesn't affect neuronal survival and there is no significant difference with control samples. All values are mean±SEM.

Both quantitative confocal microscopy and Western Blot analysis indicated that SIV-infected microglia supernatant significantly inhibited neuronal autophagy activity and this affect was reversed by rapamycin. We then sought to determine whether these changes in autophagy were involved in neuronal survival. Using the 3-(4,5-Dimethylthiazol-2-yl)-2,5-diphenyltetrazolium bromide (MTT) assay, we found that neuronal survival decreased significantly after 3 or 24 hr in the presence of the SIV-infected microglia supernatant (Figure 6A, 91.1±1.1% and 68.9±1.1% of 100% baseline, respectively). Rapamycin pretreatment conferred significant protection of the neurons against the neurotoxic effect of SIV-infected microglia supernatant after 24 hr (Figure 6A, 86.2±1.9%), although not recovering completely to the baseline level. Furthermore, pre-treatment with (5R,10S)-(+)-5-methyl-10,11-dihydro-5H-dibenzo[a,d]cyclohepten-5,10-imine (MK-801), a non-competitive antagonist of the NMDA receptor, also abrogated neuronal cell death, confirming the importance of the excitotoxic glutamate pathways (Figure 6A). Data obtained from the lactate dehydrogenase (LDH) release assay confirmed the neurotoxicity results of the MTT assay (data not shown). These data strongly indicate that impaired autophagy contributes to the neuronal damage, and enhancing autophagy with rapamycin can inhibit a significant portion of neurotoxic effect of SIV-infected microglia supernatant.

We then examined the effect of distinct stages of autophagy inhibition on neuronal cell viability using chemical inhibitors. Treatment with the lysosomal fusion inhibitor between AV and lysosomes, Bafilomycin A1 (Baf), by itself reduced neuronal survival (Figure 6B). Pretreatment with Baf prior to exposure to the SIV-infected microglia supernatant resulted in additional neuronal death, indicated that different processes maybe affected by these two agents. In contrast, treatment of cells with the inhibitor of type PI3K type III, 3-methyladenine (3-MA), reduced neuronal survival. However, a pretreatment with 3-MA followed by exposure to the SIV-infected microglia supernatant did not reveal any additional neurotoxic effect (Figure 6B).

### p62 expression in SIVE and HAD brains

**Figure 7:**
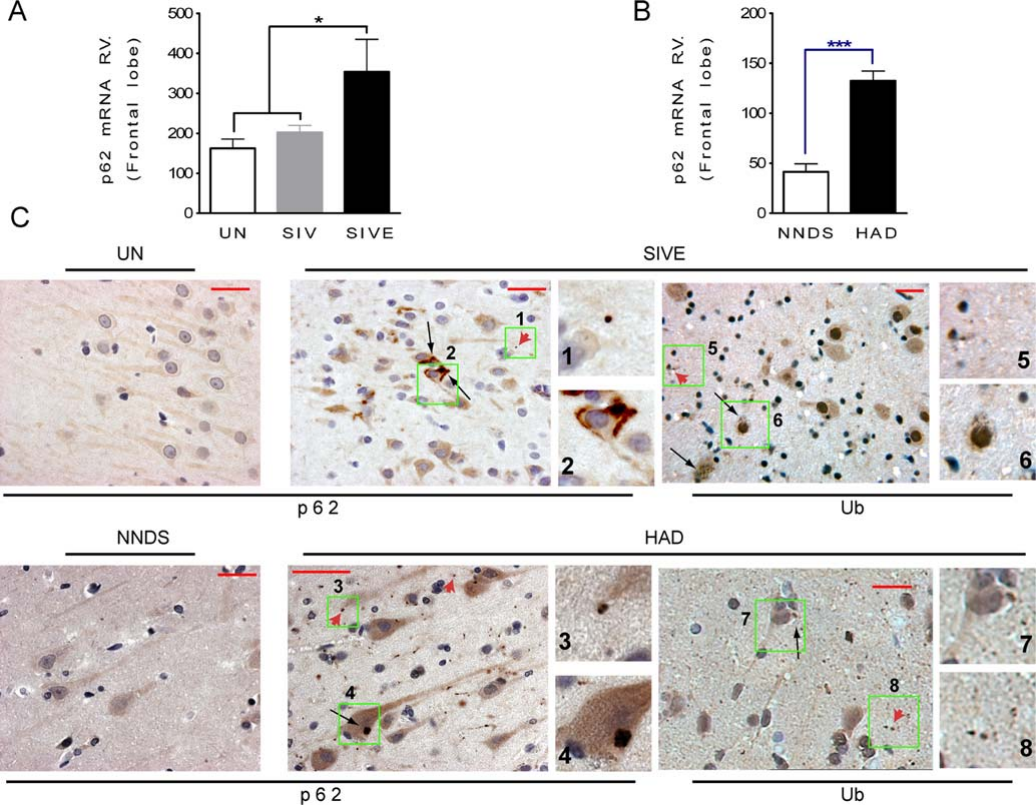
Transcriptional regulation of p62 in SIVE monkey brains, and its presence in human and monkey neurons associated with ubiquitin. (A) Relative values (RV) of p62 mRNA level in frontal lobe of SIVE versus SIV and uninfected (UN) monkeys, *n* = 7 for SIVE, *n* = 12 for SIV and *n* = 9 for UN, *P*<0.05. (B) RV of p62 mRNA level in frontal cortex of HAD versus non neurological diseased subjects (NNDS), *n* = 8 for HAD and *n* = 10 for NNDS, *P*<0.0001. (C) p62 and ubiquitin immunoreactivity on serial sections in SIVE monkeys, as well as those with HAD in brain sections from hippocampus. p62 has a diffuse immunoreactivity in UN monkeys and in human NNDS and a dot profile structures in SIVE and HAD brain tissues. Higher magnification views are shown in green insets. Black arrows indicate p62- or ubiquitin-immunoreactive structures within the neuronal cell body or proximal processes, and red arrowheads indicate a dot-like immunoreactivity in the neuropil. Scale bar, 20 µm.

It has been shown that the expression of p62 is involved in protein aggregation of different neurodegenerative diseases and its transcript is upregulated in response to pro-apoptotic conditions [35]. Moreover examining changes in p62 expression, at both the mRNA and protein levels, can be used as an index of autophagic activity [36]. To examine p62 expression, we first assessed the level of p62 mRNA in uninfected monkeys compared to those infected with SIV but lacking SIVE, as well as those with SIVE. Quantitative real time PCR analysis revealed a significant increase of the p62 transcript level (Figure 7A), shown here in the frontal lobe, in SIVE compared to both brains from uninfected animals or those infected with SIV but without encephalitis. Assessment of hippocampus, caudate and cerebellum in addition to the frontal lobe indicates an approximate 2-fold increase in p62 mRNA levels in SIVE compared to uninfected monkeys. We next examined the p62 mRNA transcripts levels in human brain specimens affected with HAD and we compared with those with non neurological diseased subjects. We found a significant upregulation of mRNA p62 level in HAD patients (3.2-fold increase) versus non neurological diseased subjects demonstrating the translation of the results from experimental animals to humans (Figures 7A and 7B). We next analyzed p62 immunoreactivity in brains of monkeys with SIVE and humans with HAD. Distinct staining within the neuronal soma and proximal portions of their processes, as well as dot-like structures in the neuropil, were found in both SIVE and HAD brains (Figure 7C). Analysis of ubiquitin antibody labeling in SIVE and HAD brains revealed a similar dot-like structures in neurons and similar abnormal pattern of increased ubiquitin immunoreactivity as was found for p62 (Figure 7C).

## Discussion

Here, we examined whether glial-derived products affect neuronal autophagy. The ensemble of imaging, biochemical and survival results strongly support the compromise of autophagy by the SIV-infected microglia supernatant. We discovered that autophagy was inhibited by exposure to SIV-infected microglia supernatant, and importantly, rapamycin treatment both enhanced autophagic activity and conferred significant protection to treated neurons. Our results identify the role of autophagy as protective in response to pro-death signaling by SIV-infected microglia in primary neurons. Furthermore, SIV-infected monkey and HAD brain tissues exhibited patterns of p62 immunoreactivity which is compatible with impaired autophagy.

Indeed, using quantitative, high-resolution three-dimensional fluorescence imaging, our experiments clearly showed that AV are significantly altered in the presence of SIV-infected microglia supernatant. Biochemical markers confirmed the imaging results. First, we found that the total AV numbers in neurons are decreased in the presence of the SIV-infected microglia supernatant, and associated with striking loss of AV number in neurites. Rapamycin treatment completely prevented this change. Consistent with these results, immunoblot experiments revealed that the key AV lipidated-protein marker, LC3-II, is also decreased in the presence of the SIV-infected microglia supernatant and the decrease is reversed with a rapamycin treatment. Further analysis showed that autophagy elongation step, which is represented by the Atg12-Atg5 complex, is severely affected in neurons exposed to the SIV-infected microglia supernatant. In addition, the accumulation of the p62 protein is consistent with the inhibition of autophagy in the presence of SIV-infected microglia supernatant. Together these results demonstrate that in neurons exposed to SIV-infected microglia supernatant, autophagy is inhibited likely at the level of induction. Both TNF-α and glutamate, leading candidates in the neuronal damage induced by CNS HIV infection, had effects similar to that of the supernatant.

Within the neurons, we find that AV are lost from the neurites. Interestingly, in neurons, microtubules facilitate AV transport, and it has been found that there is a retrograde transport of AV in the case of stress (Iwata et al., 2005). The decreased AV formation, illustrated by the decline in LC3-II and Atg12-Atg5 levels, may combine with a potential retrograde transport of AV from the neurites, leading to the detrimental affect of the SIV-infected microglia supernatant on neuronal autophagy. Whether the loss of the neurites themselves is secondary to the loss of AV or reflective of a distinct pathological mechanism requires further study.

There is convincing evidence to support the idea that autophagy has a protective function in neurons and its dysregulation is associated with distinct forms of neurodegeneration. Despite the prevalence of HAD, the molecular mechanisms underlying neurodegeneration remain poorly understood. Compounds released by HIV-infected macrophage/microglia in AIDS patients activate neuronal apoptotic death, resulting in HAD [20], [21]. Indeed in our experiments a decrease in neuronal survival paralleled the deficits in autophagy. Furthermore, survival was enhanced in the presence of rapamycin, which reversed the loss of autophagy. Although some neuronal loss still occurred, the SIV-infected microglia supernatant may trigger multiple pathways leading to neuronal death, with the impairment to autophagy constituting only one of the pathways. The mechanism by which rapamycin, *via* the inhibition of the negative regulator of autophagy, mTOR, rescues the deficit here is not known. We hypothesize that rapamycin induces autophagy at initiation step to eliminate the effect of SIV-infected microglia supernatant.

Indirect support for the autophagy defect in our experiments residing at the induction stage comes from the lack of additive effects of 3-MA in the presence of supernatant, in contrast to Baf, which acts at a late stage in the autophagy process. Moreover, pharmacological inhibition of autophagy, at early or late stages of the process with either 3-MA or Baf, respectively, activated cell death, reflecting the protective role for constitutive autophagy in primary neurons.

There is a strong interdependent relationship between autophagy and apoptosis [37]. While apoptosis is a synchronized process leading to cell death, autophagy is considered both a mechanism to protect from toxicity in stress conditions as well as a mediator of cell death. Interestingly, although autophagy can be required to induce apoptosis [38], inhibition of autophagy can also induce apoptosis [39]. Apoptosis is a major candidate process for neuronal death in the setting of HAD [40], but additional studies are needed to uncover the relationship between reduced neuronal autophagy and apoptosis in this disorder.

Therapeutic strategies based on facilitating efficient autophagy have shown promise in neurodegenerative disease models. In fact provocative experiments utilizing inducers of autophagy reveal that induction and activation of autophagy processing can cleanse intracytosolic toxic molecules or different disease-associated intracytosolic aggregate-prone proteins such as mutant Huntington and A53T α-synuclein [41]–[43]. Enhancing autophagy thus has various beneficial consequences, not only promoting the clearance of toxic aggregated proteins, but also protecting against certain subsequent pro-apoptotic insults. For example, in response to apoptotic stimuli, autophagy can remove potentially harmful mitochondria and transiently increase protective ATP levels [44], [45]. Ubiquitin-protein aggregates are also cleared through autophagy pathway and one of the characteristic of autophagy malfunction is the increase of ubiquitinated proteins, as it has been reported for different neurodegenerative diseases such as Alzheimer's, Parkinson's, and Huntington's diseases (AD, PD, and HD) as well as the genetically deficient *Atg5* and *Atg7* knockout mice.

It has been found that the increased expression of p62 secondary to impaired autophagy may predispose to inclusion formation [46]. Interestingly, it has recently been demonstrated that ubiquitin-positive inclusions, which were otherwise abundantly present in Atg7-deficient neurons, are markedly reduced by genetic deletion of p62 [18]. In neurodegenerative diseases increased p62 is a common feature detected in inclusion bodies containing polyubiquitinated protein aggregates, including Lewy bodies in PD, neurofibrillary tangles in AD, and Huntingtin aggregates in HD [14], [47], [48]. The increased transcript level of p62 found here may suggest that it can be implicated in physiopathology of protein aggregation and/or it is a natural response to the pro-inflammatory conditions occurred in these infected subjects. Decreased neuronal autophagy could lead to increased p62 and ubiquitinated protein immunoreactivity within the neurons/neuropil, as we saw in SIVE and HAD brains. This result is further supported by reports of others of ubiquitin protein deposits in neurons in those with HAD [27]. These findings are consistent with recent report that the autophagic removal of ubiquitinated protein aggregates is mediated by the interaction between p62 and K63-linked polyubiquitin [49].

Here we clearly show that pathological glia can induce the perturbation and decrease of autophagy in neurons, leading to neuronal demise. Autophagy can be considered as the verve of non-dividing cells such as neurons. Homeostasis of autophagy is be a reliable security factor which needs to be maintained and protected in neurons exposed to any insults, such as those leading to neurodegeneration diseases, demonstrated here for the SIV model of HAD.

## Materials and Methods

### Neuronal cultures, transfection and drug treatments

Primary neurons were cultured as previously described [50], [51]. In brief, the cerebrocortices of Sprague-Dawley rat embryos at day 17 of gestation, were digested and plated on poly-DL-lysine (30–70 kDa, Sigma-Aldrich, St. Louis, MO, USA) coated glass coverslips at a density of 3×10^5^ cells/ml in high glucose Dulbecco's Minimum Essential Medium (DMEM) containing GlutaMax™ supplemented with 10% bovine calf serum, 25 mg/ml penicillin/streptomycin. The cultures were incubated at 37°C humid incubator for 60 min before switching to a neurobasal medium containing 2% B27 (Invitrogen, Carlsbad, CA, USA). Fluorodeoxyuridine (10 µM; Sigma-Aldrich) was added 1–3 days after plating, and cells were fed twice weekly thereafter. All cells were grown at 37°C and in 5% CO_2_. Most of the experiments were performed on 16–20 days *in vitro* cultures unless otherwise indicated. The purity of the neuronal population was verified by immunocytochemical staining for MAP-2. Under these conditions, the cultures were >98% homogeneous for neurons.

Cells on coverslips were transiently transfected at 12–17 divisions with 0.5 µg of GFP-LC3 plasmid DNA [10], using Lipofectamine 2000 reagent (Invitrogen). Under these conditions, <2% transfection efficiency was achieved in neurons and experiments were performed after 2–5 days.

Transfected cells on coverslips, or cells grown on 24 well plates for MTT and LDH assays, or 6 well plates for Western Blots, were treated, for the indicated times, with supernatant from SIV-infected at a final concentration of 1∶20, or control uninfected, microglia. For drug treatments, cells were treated with rapamycin (2 µM, EMD Calbiochem, San Diego, CA), TNF-α (25 ng/ml, PeproTech, Inc., Rocky Hill, NJ), NMDA (35 µM, Sigma-Aldrich), MK-801 (100 µM, EMD Calbiochem) 3-methyladenine (1 mM, Sigma-Aldrich), or Bafilomycin A1 (100 nM, Sigma-Aldrich), for 30 minute prior exposure (or not) to the supernatant from SIV-infected microglia.

### Three dimensional confocal AV Counting Assay

Images were acquired using a Rainbow Radiance 2100 Laser Scanning Confocal system attached to a Nikon TE2000-U inverted microscope (BioRad-Carl Zeiss Inc., Thornwood, NY, USA). All images were 8-bit optical slices and were obtained using a 60× (1.4 N.A.) objective to capture the entire neuron (cell body and processes) at the best resolution (0.2 µM interval step slices, Figures 1A–1J). Images were acquired using Laser Sharp 2000 software then imported and further analyzed for detailed quantitative 3D analysis using the IMARIS software package (BitPlane Inc., MN, USA). Once imported into the IMARIS software, the macro “filament tracker” was used to automatically trace the GFP-LC3 fluorescence signal in 3D space to define the spatial location of the cell body and all of its branches, dendrites and axon (Figures 1K–1O), and thus create a 3D outline of the neuron (macro designed for this purpose in IMARIS). For this process, to outline and define the entire neuron, a larger and less discriminating fluorescent threshold range was used (between 10–256 shades/levels of grey), which includes specific labeling and cytoplasmic haze that defines all the branches. Once this detailed outline of the neuron was created (which has dimensions that are based on the original signal), the software can extract parameters such as neurites area and volume, number of free unassociated termination points and number of branch points.

Alternatively, a very discriminating signal threshold range (between 100 and 256) was used to define a real signal above background for outlining and marking positively labeled vacuoles. This range was used to define the GFP-LC3 vacuoles through out the cell body and its branches (axon and dendrites). In addition, we determined that the size of the smallest vacuole that can be distinguished as a vacuole by the software successfully to have an average diameter of 1 µm. The “spot tracker” tool in IMARIS was then used to automatically track this thresholded signal range and defined diameter per vacuole in 3D space and place a sphere at the centroid of each bright signal spot that was deemed a vacuole (macro designed for this purpose in IMARIS, see Figure 1B). Once this was accomplished, the IMARIS software calculated parameters such as the number detected, and their fluorescent intensity and location. The data was then exported to MS Excel and further analyzed for statistics and graphing. For the various conditions described above at least 200 cells were used over 8 experiments.

### SIV-infected Microglia Supernatant Preparation

To mimic the indirect effect of HIV/SIV on neurons, we prepared supernatants from microglia/macrophages derived from the brains of SIV-infected and uninfected rhesus monkeys (Figure 2A). All animal work was performed under NIH guidelines with the approval of the TSRI Institutional Animal Care and Use Committee. For monkey infections, a microglia-passaged stock of SIVmac251 was used, denoted SIVmac182, obtained by 3 generations of *in vivo* serial passage [52], [53]. Brains were obtained from SIV-infected or uninfected monkeys following vascular perfusion with PBS/heparin at necropsy. Tissue was disrupted, digested with collagenase, and microglia/macrophages isolated by a Percoll gradient technique [54]. Cells were plated in 6 well plates at a density of 3×10^6^ cells/ml in RPMI 1640 containing glutamine supplemented with 10% bovine calf serum, 25 mg/ml penicillin/streptomycin, 50 ng/ml Macrophage Colony Stimulating Factor (PeproTech, Inc.) and cultured for up to 30 days. Following the first 7 days of culture of cells from SIV-infected animals, the supernatant was harvested daily, and the level of SIV p27 Gag measured by ELISA kit. Those with p27 levels >1 ng/ml were pooled and centrifuged through Centriplus filters (Millipore Billerica, MA, molecular weight cutoff 30 kDa) and filtrates (<30 kDa fractions) collected. The equivalent days post-plating were collected from cultures derived from uninfected monkeys as control supernatants.

### Brain tissues

The National NeuroAIDS Tissue Consortium (Rockville, MD) provided clinical diagnosis and post-mortem brain tissues from 8 individuals with HAD, and 10 individuals without HIV infection and without any neurological symptoms or significant neuropathology. Tissue from 7 uninfected rhesus monkeys and 8 with SIVE were from our previous studies. All samples were obtained under institutional regulatory approval following NIH guidelines.

### Immunofluorescence statining

After different treatments, cells were fixed for 25 min with ice-cold 4% paraformaldehyde (PFA) at 4°C and washed three times in PBS, at room temperature. PFA-fixed cells were permeabilized by using 0.2% Tween 20/PBS, and nonspecific binding sites were blocked by incubation for 1 hr with a 10% solution of heat-inactivated goat serum in 0.2% Tween 20/PBS. To specifically stain neurons, cells were then incubated for overnight at 4°C of anti-microtubule associated protein-2 (MAP-2; Sigma-Aldrich) at 1∶500. Cells were then incubated with secondary polyclonal antibody, Rhodamine Red™-X conjugated goat anti-mouse IgG (Invitrogen) at 1∶500 for 1 hr at room temperature. Cells were counterstained with the fluorescent nuclear stain 4′6-diamidino-2-phenylindole (DAPI; Sigma-Aldrich) at 1 µg/ml, air-dried and mounted on glass microscope slides in Vectashield mounting media (Vector Laboratories, CA, USA).

### Western Blot analysis

Following different treatments, neurons were washed one time in ice-cold PBS, scraped, and lysed in ice-cold RIPA lysis buffer [50 mM HEPES buffer, 1 mM sodium orthovandate, 10 mM sodium pyrophosphate, 10 mM NaF, 1% NP-40, 30 mM *p*-nitrophenyl phosphate (MP Biomedicals, Solon, OH, USA), 1 mM PMSF, 1× complete protease inhibitor cocktail (Roche Diagnostics, Indianapolis, IN, USA)]. After incubation for 30 min on ice, samples were centrifuged at high speed for 25 min and supernatants containing soluble proteins were collected. Total protein concentration was determined with the bicinchoninic acid assay, using bovine serum albumin as standard. Proteins were separated on NuPAGE 12% (for LC3 antibody) or 4%–12% Bis-Tris acrylamide gradient gels (Invitrogen) and transferred onto electrophoretically to HY-bond™ PVDF membranes (Invitrogen). Nonspecific antibody binding was blocked by either 5% BSA or nonfat dried milk for 1 hr at room temperature. Immunoblotting was carried out with antibodies against LC3 (MBL International, Woburn, MA, USA), Atg5 [8] and p62 (BD Transduction Laboratories, San Diego, CA, USA), followed by secondary antibody (1∶10,000 HRP conjugated anti rabbit or anti mouse IgG; GE Healthcare, Little Chalfont, UK). Blots were developed with 1∶1 solution of Super Signal West Pico Chemiluminescent Substrate and Luminol/Enhancer (Thermo Fisher Scientific, Rockford, IL, USA). Blots for loading control were stripped subsequently using ReStore® Western Blot stripping buffer (Thermo Fisher Scientific) then re-probed for GAPDH (Millipore, Billerica, MA, USA). The optical density of bands was quantitated using the ImageJ v.1.38 software and the ratios to GAPDH were normalized for time 0 hr and then expressed as fold- values of the mean±SEM.

### Reverse Transcription and Quantitative Real Time PCR

Isolation of RNA and quantification of specific mRNAs by real-time PCR in brain specimens was performed as described previously [55]. For rhesus monkey and human p62, primers were CAGTCCCTACAGATGCCAGA and TCTGGGAGAGGGACTCAATC, the double-labeled probe was TCCCAGGAGGGACCCACAGG. The average of 18S, TATA box binding protein (TBP) and glyceraldehyde 3-phosphate dehydrogenase (GAPDH) RNA were used for macaques, and 18S and GAPDH for human, as the endogenous controls using the primers/probe sets described [55].

### Immunohistochemistry

Formalin-fixed, paraffin-embedded of serial brain sections were cut at 6 µm thickness, mounted on glass slides, deparaffinized and subjected to heat-mediated antigen retrieval (AR) with a steamer for p62 and ubiquitin respectively. Indirect immunohistochemistry was then performed as previously described [55]. Primary antibodies were a mouse monoclonal antibody against p62 (BD Transduction Laboratories), using pH 9 Tris-EDTA buffer for AR, and rabbit polyclonal antibody against ubiquitin (Lab Vision Corp., Thermo Fisher Scientific, Fremont, CA), using 0.01 M citrate buffer, pH 6.39 for AR.

### Neuronal Survival and Toxicity Assays

Cell survival and death were measured conjointly in 24-well culture dishes 24 hr after the indicated treatments. Neurotoxicity was monitored by the measurement of LDH activity released from the cytosol of damaged cells into the supernatant. Culture medium (200 µl/well) was sampled and centrifuged to remove cellular debris from the supernatant in order to measure the LDH enzymatic assay following the vendor's kit manual (Roche Applied Science, Indianapolis, IN). Triton X-100-treated (2%) cells were used to determine the maximum releasable LDH activity in the cells. The percentage of LDH release was calculated as LDH in the culture medium divided by total LDH (maximum releasable LDH activity). For the assay, LDH assay reagent was incubated for 30 min at room temperature, and the absorbance of samples was measured using a Victor3 Multilabel Counter (PerkinElmer, Boston, MA), taking absorbance measurements at wavelength of 490 nm. The same plate was then used to monitor the mitochondrial respiration using the reduction of MTT into a blue formazan precipitate. For the MTT assay, neurons grown in 24-well culture dishes were incubated with MTT (0.5 mg/ml) for 30 min at 37°C. The blue formazan produced was solubilized in 1 ml of dimethyl sulfoxide, and the amount of MTT formazan product was determined by measuring immediately using a Multilabel Counter 1420 Victor (PerkinElmer), taking absorbance measurements at wavelength of 560 nm.

### Statistical Analysis

Statistical analysis was carried out using GraphPad Instat. All experiments were repeated at least six times, unless otherwise stated. Results are shown as the mean±SEM. For two groups data were evaluated by Student's *t*-test, for more than two a one-way analysis of variance (ANOVA) followed by *post-hoc* Tukey's test was used for multiple comparisons. Significance was assessed at *P*<0.05.
